# Perceptions of multiple chronic conditions and coping strategies among migrants from Sub-Saharan Africa living in France with diabetes mellitus and HIV: An interview-based qualitative study

**DOI:** 10.1371/journal.pone.0284688

**Published:** 2023-06-02

**Authors:** Soline de Monteynard, Hélène Bihan, Lucie Campagné, Cyril Crozet, Johann Cailhol

**Affiliations:** 1 Department of Infectious and Tropical Diseases, Avicenne University Hospital, Bobigny, France; 2 Endocrinology-Diabetology Department, Avicenne University Hospital, Bobigny, France; 3 LEPS UR 3412, Sorbonne Paris Nord University, Bobigny, France; 4 Department of General Medicine, Sorbonne Paris Nord University, Bobigny, France; Faculty of Health Sciences - Universidade da Beira Interior, PORTUGAL

## Abstract

Although diabetes is common among people living with HIV/AIDS (PLWHA), few data exists on how migrants from Sub-Saharan Africa (SSA) experience living with these two coexisting conditions in France. The objective of this study was to analyze perception of polypathology among PLWHA from SSA with type 2 diabetes and identify barriers and facilitators to their self-management. A qualitative study was conducted using semi-structured interviews from November 2019 to April 2020 with participants selected from a cohort of PLWHA and diabetes at Avicenne University Hospital. A total of 12 semi-structured interviews were conducted and analyzed using thematic analysis with inductive approach. Stigma remained a major issue in self-managing HIV, and some participants did not consider themselves as having a polypathology, as HIV has always been considered as a distinct condition. In general, emotion-based resources (e.g spirituality, trust in the medical discourse) and social support were mobilized more than problem-solving resources (e.g perception of medication as life-saving). Participants used the same main resource in self-management of HIV and diabetes, and resources used differed from participant to participant. This study highlighted challenges in self-management of diabetes and HIV in this population and complexity related to the socioeconomic and cultural specificities. Self-management could be more successful if patients and carers move in the same direction, having identified the individual coping resources to reach objectives.

## Introduction

In France, nearly 6200 people discovered their HIV status in 2018, most of whom were born abroad (56%); among those born abroad, 66% were born in Sub-Saharan Africa (SSA) [1], reflecting the high proportion of migrants from SSA in France (47.5% as of 2021) [2]. Usually, many migrants from countries with limited resources, especially from SSA, encounter difficulties, in both the migration process and reception in the host country [3]. Despite relatively good health at the time of leaving their country of origin, this population is considered to be at high risk of developing health-related problems over time because of their irregular status and the effects of economic and social marginalization [4, 5]. Reportedly, socioeconomic conditions and access to health rights are the main risk factors for medical vulnerability in this population [6, 7].

Concerning HIV, migrants are at a higher risk of delayed diagnosis of HIV infection [8]. Indeed, 35% of migrant women and 45% of migrant men are diagnosed with advanced HIV disease (CD4 count below 200 cells/mL or AIDS-defining event) [9], and migrants account for 46% of people diagnosed with AIDS [10]. Late presentation, which may reflect late diagnosis and/or late entry into care, has consequences for the individual, in terms of poorer outcomes and mortality [11].

Universal access to antiretroviral therapy (ART) has remarkably decreased HIV-related mortality and increased the life expectancy of people living with HIV/AIDS (PLWHA) [12, 13]. Once associated with opportunistic diseases (infections that occur more often or are more severe in people with weakened than in people with healthy immune systems such as tuberculosis, toxoplasmosis…), HIV-related mortality is currently attributable to age-related comorbidities, including dyslipidemia, hypertension, and type 2 diabetes. Thus, half of the deaths of PLWHA are related to a non-AIDS cause, primarily cardiovascular [14]. However, several studies in the United States, Europe, and Africa have also demonstrated that ART also plays a role in the development of lipodystrophy, dyslipidemia, and diabetes [15, 16]. Depending on the study, the prevalence of diabetes among PLWHA varies between 2% and 14% [17, 18]. Along with multiple diseases, significant socioeconomic disparities exist in the occurrence of diabetes and its complications. A low socioeconomic level is a risk factor for developing diabetes with related consequences [19–23]. Thus, many migrants from SSA living with AIDS are now living with a polypathology, HIV and diabetes.

The emerging concept of polypathology is complex and broadly defined as the existence of two or more chronic conditions in the same individual [24].

Therapeutic patient education or self-management education [25] makes the patients an actor in their care by acquiring self-care and coping skills, thereby supporting patients with multiple diseases using programs that include educational tools or materials. In particular, it helps patients to fortify their coping strategies, as described by Lazarus and Folkman [26]. The term “coping” describes the actions that the patients develop to control, reduce, or tolerate difficult, stressful, or incomprehensible situations [27]. Thus, improving the self-management education of patients with polypathology warrants a better understanding of the barriers and facilitators. Some previous studies listed the main barriers of self-management as financial, logistical, and physical constraint, lifestyle changes, psychological consequences, poor family and social support; drug use; and the relationship with the healthcare system [28, 29]. On the side of the care system, facilitators of self-management have been identified as follows: health mediation, ethnopsychology, and care coordination [30].

Although cultural factors should also be considered, research on self-management in polypathology in populations of African origin has been conducted in the United States or the UK. To the best of our knowledge, no related study has been conducted in France. The majority of PLWHA in France are from SSA, and many have multiple diseases (especially diabetes); however, no study in France has explored the self-management of polypathology among PLWHA. Hence, this qualitative study aims to explore the perception of polypathology among PLWHA from SSA with type 2 diabetes and identify barriers and facilitators to their self-management.

## Materials and methods

### Study design

Given the exploratory nature of this study, without any pre-specified hypothesis, a qualitative design, allowing an inductive approach was considered as the most appropriate. The inductive approach was used in order to identify new themes emerging from the participant’s discourse. The study was conducted in a hospital setting (Avicenne University hospital), given the health system characteristics in France, where PLWHA are mostly followed-up in large hospitals. The geographic area where the university hospital is located is the most deprived area of metropolitan France, with a large proportion of migrant population among the inhabitants. Indeed, the Avicenne Hospital is located in the department of Seine Saint Denis where nearly 30% of the population is immigrant [31]. The poverty rate in this department is the highest in metropolitan France (27.9% in 2015) [31].

### Study population

We first selected participants from the PLWHA cohort followed up at the Avicenne Hospital using the Nadis software ® (ABL, Paris, France, version 1.1.498.4), a specific HIV electronic health record. All participants matching the following inclusion criteria were considered eligible: aged >18 years; born in SSA, HIV infection (type 1 or 2) controlled or not; type 2 diabetes diagnosed after the HIV diagnosis and treated either with oral antidiabetic drugs or by insulin therapy, controlled or not; fluent in French; and willing to participate voluntarily in the study. The exclusion criteria were as follows: diabetic participants on a diet alone, non-consenting participants, and totally non-French nor English-speaking participants.

Diabetes was defined as controlled for any HbA_1c_ < 7% [32]. HIV control was defined as an undetectable HIV-1 RNA plasma level (< 40 copies of HIV per milliliter of blood) for at least 6 months. We chose as a criterion a diagnosis of diabete subsequent to that of HIV to study polypathology through the prism of HIV as an entry into the disease and to respect the same temporality between the two diagnoses. There was no a specific duration of time that the individual had to have had HIV and/or diabetes. We excluded diabetic participants on a single diet to assess the impact of polymedication in the self-management of polypathology.

### Interview guide

The interview guide was built as to explore the temporality of diseases discovery, the coping strategies in HIV history and in diabetes history separately and explored the notion of polypathology.

The interview guide was first pilot-tested with one participant and further adapted as the interviews were transcribed and analyzed. Between analysis and data collection, the process was continuous, and the discovery of new elements in an interview led to the modification of the guide for subsequent interviews. S1 Annex presents the latest version of the interview guide used in this study.

### Data collection

The investigator contacted all the eligible participants telephonically. The study and the interview process were briefly presented to the potential participants. Then, the place and date of the interview were decided by the participant (home or hospital).

A follow-up telephone call was made 24 h before the interview. The interviews were recorded after obtaining the oral consent from participants via a voice recorder on the investigator’s personal phone. There was only one interviewer (the investigator), a white woman, who was trained as a physician and by her research supervisor (PhD) in performing semi-structured interviews. There was no prior relationship between interviewer and participants. The interviewer introduced herself as a doctor completing a research for improving care in HIV/diabetes polypathology. Only the investigator and participants were present during the interview.

Of note, participants were recruited until data saturation, which was discussed after each interview between the interviewer and supervisor and verified also with subsequent participants [33].

### Data analysis

All interviews were transcribed using Microsoft Word software. The data were subjected to thematic analysis [34], with an inductive approach performed in parallel with the interviews to determine the end of recruitment when data saturation was attained. The interviewer performed the whole data analysis by manual open coding. It relied on the detailed reading of the raw data to bring out categories. In addition, double coding, with the help of the research supervisor, was done for two transcripts to increase the internal validity of the study. Then, an axial coding and finally a selective coding were performed by the researcher and the supervisor in order to identify the main themes presented in this paper. We identified first semantic themes and deepened the analysis towards latent themes. We followed COREQ guidelines for data analysis and data collection (S1 Checklist).

### Ethics

The Inserm Ethics Review Committee (IRB00003888, IORG0003254, FWA00005831) gave a favorable opinion for this project. Participants were informed orally of the study objectives, and their participation in the interview constituted consent. They were informed that their answers would be anonymized, that they could withdraw their consent at any time, that not answering a question would not affect their care, and that they could access the study results.

The ethics committee specifically asked for waiving the written consent procedure, due to the vulnerable nature of this category of population. The oral consent was considered as obtained when the recording started, *as per* ethics committee’s instructions.

Anonymity was respected by omitting from the record any nominal data that might allow the identity of the participant to be recognized. After transcription, each interview was deleted from the investigator’s mobile phone, and each anonymous file was numbered with a code from M1 to M12.

No participant expressed interest in being contacted again for providing study findings or reading the transcripts, probably given that few were able to read.

## Results

### Participants’ characteristics

The interviews took place between 22^th^November 2019 and 5^th^ January 2020. In total, 27 eligible participants were called, of whom 12 accepted the interview (see Table 1). All interviews were conducted in French. There was no repeat interview. The interview duration varied according to the personal time available to each participant and the ease with which they could share their experiences (one hour on average). Median age was 54 years old. The reasons for refusal were various (lack of time, poor understanding of French, and departure abroad). The range of year of arrival in France was between 1980–2007. Of all, 11 participants had controlled HIV infection, and 6 had controlled diabetes. Regarding the country of origin, three participants were from the Democratic Republic of Congo, three from Mali, three from Cameroon, one from Congo-Brazzaville, one from Côte d’Ivoire, and one from Senegal. Data saturation was reached after the 11th interview and we confirmed this hypothesis with the 12^th^ interview during which no new information emerged. The analysis of the study yielded over 500 codes, which were then grouped into subthemes, themes, and then more general topics. We identified several themes and divided those into four categories: representation of two diseases (origin, perception of their severity, resources mobilized); self-management of the polypathology; and experience of the care pathway since the polypathology.

**Table 1:** Participants’ characteristics.

M	Gender	Age	Location of the interview	HIV discovery	Diabetes discovery	Controlled HIV	Controlled diabetes
**M1**	M	40	Hospital	2002	2015	YES	YES
**M2**	F	54	Hospital	2002	2009	YES	YES
**M3**	F	72	Home	2003	2013	YES	YES
**M4**	M	44	Hospital	2006	2013	YES	**NO**
**M5**	F	60	Home	1999	2015	YES	**NO**
**M6**	M	70	Hospital	2002	2006	YES	**NO**
**M7**	F	53	Hospital	2004	2006	YES	YES
**M8**	F	65	Home	1998	2019	YES	YES
**M9**	F	53	Hospital	2003	2003	YES	YES
**M10**	F	54	Home	2001	2019	YES	**NO**
**M11**	M	60	Hospital	2002	2005	YES	**NO**
**M12**	M	47	Hospital	2004	2014	**NO**	**NO**

### The long road to polypathology

#### HIV: A disease of death and shame

The context of the discovery of HIV for many of our participants was still very clear in their memory, despite the time elapsed since the diagnosis. For many participants, this discovery took place after their arrival in France and was a real shock.

**M3:** *It gave me tension*. *I was very sick and it threatened me*. *We were all very affected*. *It was a shock*, *I was afraid*, *I cried*, *all week my daughter gave me food I didn’t eat*. *She herself was crying*.

Many participants spoke of HIV as a disease where death is the only possible outcome.

**M2:** *Because every day my family cries*, *they cry*, *they pray*. *I thought I had a few days*, *a few months to live*. *I even asked the doctor how long I have to live*.

Besides death, some also referred to HIV as a disease of the mind, an invisible disease, an imaginary disease because it is invisible and occasionally asymptomatic and because it does not exist in the minds of some of their compatriots.

**M2**: *When the disease started in Cameroon*, *we talked about condoms and everyone didn’t believe in it*.

In some African cultures, blood also represents energy and spirit [35]). As HIV circulates in the blood, the representation of HIV as an invisible disease and an invisible spirit is also present.

**M6**: *In fact HIV is like the mind*, *because it’s in the blood*.

Shortly after confirming the seropositivity, the evocation of the cause of the disease and the search for it was presented in all the interviews. Questions included “why me?” and “how did I get infected?” One participant referred to HIV as a “disease of shame.” This shame was also found in identifying HIV with “immoral” behavior such as infidelity, relationships with prostitutes, or a high number of partners. Other, less guilt-inducing modes were also cited, such as a transmission from the hairdresser back in their country of origin, or a curse. It was important in all interviews that the cause was outside their responsibility.

#### The onset of diabetes: Represented by heredity and amputation

Unlike that of HIV, the diagnosis of diabetes was not received with a shock by most of our participants; this diagnosis was mainly done by an infectious disease specialist. In addition, the comparison with HIV was very present. The diagnosis of HIV before that of diabetes could lighten the burden of the diagnosis of this second chronic pathology.

**M11:** *I didn’t react*! *Good diabetes there*, *there are a lot of people who have diabetes*, *it was AIDS that scared me more than diabetes*.

Sometimes, when the diagnosis of HIV was a shock, the diagnosis of diabetes reactivated this old trauma. The case of M12 illustrates the succession of shocks. M12 confided that the second ordeal he was currently experiencing was the diagnosis of diabetes, which caused him to sink into depression. The diagnosis of HIV was the first shock that required substantial energy to mobilize his resources to cope. The diagnosis of diabetes came next like a stab wound in his life. The energy needed to draw on these resources was absent to overcome this new shock, leading to discouragement in the management of his two diseases. Following the diabetes diagnosis, he stopped taking his HIV treatment during a limited time following the shock period.

Compared with HIV, etiology of diabetes is primarily perceived as “hereditary”, a family disease with high frequency, notwithstanding the type 1 or 2 of diabetes.

**M7:** *I didn’t ask any questions because I saw that it’s hereditary*. *My father has it*, *my grandfather has it*, *so I didn’t give it much thought*. *I already had a big problem*. *I trivialized it as I saw that it’s a family thing*.

Despite being hereditary, uncontrolled diabetes is often associated with member amputation, with severe consequences that cannot be controlled by participants themselves. Perhaps the seriousness of diabetes is related to their representations of the physical consequences, unlike that of HIV, which is associated with the societal consequences. Hence, diabetes is a disease that destroys the body, whereas HIV could be a disease of the “spirit.”

#### Different notions of polypathology: “being sick” or having HIV and diabetes

We observed two attitudes: (i) the clear distinction between the two diseases, HIV and another disease (e.g., hypertension and diabetes), and (ii) the overall feeling of “being sick” by combining the two pathologies inclusively. Indeed, for those who reported a clear distinction between HIV and diabetes, the source of the distinction was the stigma attached to HIV “the disease of silence, the disease of shame” and the others.

**M1:** *Even if it’s more serious than HIV it’s not the same*. *Because HIV is considered a disease of shame*. *If you tell someone that they are diabetic they don’t mind*, *but if I tell you that I have HIV*, *they look at me differently*, *it changes*. *It’s not the same*.

Thus, no disease can be compared with HIV. For those living in hiding with HIV, the diagnosis of diabetes could be the origin of the acquisition of social legitimacy as a “patient.” For others, the main aspect seemed to be “being sick.” Irrespective of the number of diseases, their names, and their consequences, the importance given to each of them specifically did not seem to vary; whether one is “monopathological” or “polypathological,” the important thing is to enter the pathology.

**M5:** *All diseases*. *Because sometimes with blood pressure you get tired*, *with diabetes you get tired*, *with HIV you get tired*. *All illnesses are tiring*. *I had pain here*, *he says it’s arthritis*. *This disease takes hold of you*, *you say this is what will kill me*. *All diseases are the same for me*.

Regarding severity, for some participants, the most serious illness was diabetes, while for others the severity was equivalent. The reasons for the seriousness of diabetes were in the consequences that it could cause, some of which are unpredictable and uncontrollable. Moreover, this seriousness correlated with the representation of diabetes as a disease that is challenging to control with medication alone, unlike HIV.

**M4:** *Where we are*, *diabetes is the most serious*. *Why is that*? *Because HIV can be controlled*, *and I am convinced that in a few years’ time there will be a vaccine for it*, *curative or preventive*. *But diabetes*, *once it’s there*, *it’s settled*.

For those of equivalent severity, they expressed the severity of “being sick” and not the name of the disease(s).

**M12:** *Both are serious*. *There is no disease less serious than the other*, *all diseases are serious*, *whether it’s cancer*, *diabetes*, *HIV*, *everything*.

### Self-management

#### An identical main resource for both diseases

In the self-management of their diseases, participants most often used a single main resource which was the same for HIV and diabetes.

These resources differed from participant to participant, but all mentioned one main resource. The main resources mobilized were as follows: personal (need to be strong and can only rely on oneself), family (living for and through one’s family), spiritual (God is the only savior), professional (through socioprofessional integration), and medical (medication and doctors).

**M12:** *Yes*, *yes*, *but it takes time*. *It already took me a long time to accept the first illness*, *and then on top of that I was told I had a second one*. *It takes time*, *but as I’ve always said*, *my mother lives*, *I love her very much*. *It takes time*, *but maybe I’ll be able to get over it with this*.

The personal resource was the need to “be strong” because the fight is “in the head.” They knew it was their responsibility to control these diseases and, thanks to the drug, these were in fact controllable, which reinforced their own responsibility. The family resource was also widely used in their experience of these pathologies. The family was a resource in terms of both sharing the diagnosis, which eased its burden, and the objective of living for the family.

Whatever the religion, “God” was a major player in their care. “God” may decide on their illness, as the provider of “fate,” or God as a guide giving knowledge to doctors. The belief in fate seemed to be a source of undifferentiated treatment of one disease or another, as “fate is responsible for everything on earth.” This removed any source of emotion regarding the disease(s). Nevertheless, the relationship with religion did not seem to be spared from the fear of stigmatization and rejection in their management of HIV. Thus, some participants maintained their relationship with their religion in isolation, preferring to pray alone at home. Hence, comparison of the resources used for HIV and diabetes revealed that all participants used the identical main resource for both, although this so-called ‘transfer’ was not explicitly expressed by these participants.

#### Science that saves

The medical resource is a widely cited and used resource by participants For HIV, as for diabetes, doctors and medicine were considered saviors. What is important is to follow the rules imposed and assess the impact on one’s quality of life. Adherence to and respect for “the rules” set by the doctor is a priority, which, if attained, ensures well-being. These rules seemed to be crucial and constitute a framework they should delimit the boundaries of the good balance of their chronic pathology. Only the hospital doctor was mentioned, and we note the absence of any other healthcare practitioner’s name from the testimonials.

The main resource mobilized and made explicit in both diseases was the representation of the role of medication and the trust placed in the medical world. The role of medication as a potential savior was transmitted from the experience of HIV to the management of diabetes by several participants. The use of this main resource in both conditions seemed to be more easily made explicit by the participants compared with the wider use of the same resource in this condition.

**M2:** *He told me we found that you have diabetes too*. *I said*, *“yes there is a tablet*.*” I said no worries*, *I’ll mix it with the others*.

Like the positive, salutary representation of medicine, the relationship of trust in the medical world seemed to be common to the self-management of the two pathologies. By following the doctor’s rules and prescribed medicines, the disease seemed to be fought well.

**M5:** *All the illnesses I have*, *I took this*. *No one can cure me if it’s not the good Lord but I have hope in my doctors even they can’t cure me but I have to respect their rules afterwards we can move on*.

When multiple providers were present, no participant was upset by some contradictions between practitioners, which is also not reported in the literature (but with different study populations) [24, 29, 36, 37].

**M3:** *Even if they give me ten doctors*, *I will take for my health*, *even twenty*. *When you pronounce the name of the doctor*, *it’s like pronouncing the name of God*. *Even the doctor who operated on me*, *whatever they give me I take*. *They themselves love me*.

General satisfaction was reported by all participants, considering that they were well taken care of for their polypathology.

#### Special case of Non Governmental Organization (NGOs) resource: Unused resource for diabetes

Among the resources mobilized in HIV self-management, several participants mentioned NGOs as a help in the experience of their pathology.

**M2:** *We give you a little coffee in the morning*. *If noon finds you*, *we give you food*. *It was good for me*. *And the social worker helped me with the papers*.

For HIV, these organizations are places where patients can learn about the disease, meet other people, and share their experiences. NGO resources were cited as a place to meet people and combat the feeling of loneliness that they might experience after the diagnosis. NGOs reassured participants, who knew that they could find support in case of need, explanations, and possible help in talking to care. This resource was seemingly used a lot at the beginning of their care and then more occasionally afterwards.

**M7:** *There was the NGOs next to Saint-Denis*, *Ikambere*. *We went there*. *We cooked*, *we discussed*, *we ate together*. *They showed us how to make medicines*, *how to sew*. *I went two or three times*, *then we met here*, *then it was fine*.

For some participants, this resource was often inspiring and a source of commitment. Being committed to others and being involved in the fight against HIV is also fighting for yourself. Many of our participants were committed to helping other PLWHA through their experience. All the participants were offered this resource in the context of HIV, which testified to the awareness of health actors of this resource; this support, far from being in opposition to the participants’ family, strengthened it and restored the links between participants and their entourage. If the objective was attained, participants became members of this new family. Unlike in HIV, NGO resources were absent in diabetes in various interviews.

#### Barriers to self-management

The barriers encountered in the management of HIV and diabetes highly correlated with the individual representation of each disease individually. In the case of HIV, four main barriers were identified: (i) physical barrier related to the modification of one’s own body image; (ii) drug barrier related to the hidden use of ARVs (concealing pills/pill bottles from others), their number, and side effects; (iii) personal barrier in the acceptance of the diagnosis and the resulting psychological consequences; and (iv) social barrier through stigmatization in all areas of their lives.

Through the representation of HIV as a disease of shame, stigma was reflected in all the barriers to self-management of this disease. Self-stigmatization contributed to the modification of one’s own physical representation, and stigmatization to the hidden use of ARVs and the personal barrier of accepting this diagnosis of shame. One of the major difficulties encountered in HIV self-management was the exclusion experienced after the announcement of HIV status.

**M8:** *I was staying with my half-sister and then it didn’t work out when she saw that I had AIDS*, *it caused problems*. *She had given me a room at the beginning*, *then she said the room there*, *she was going to give it to her daughter*.

Exclusion and stigmatization were experienced not only in the family circle but also in the social sphere. The fear of a change in the way others looked at them (experienced or imagined) and of societal exclusion was very often the first subject mentioned when asked about the main difficulties experienced.

**M2:** *I used to say to God “what am I going to do*, *what am I going to do*?*” At that time when you tell someone you are HIV-positive*, *they would immediately kick you out of their house*. *They don’t want to mix with you because before we used to say that you shouldn’t use the same spoons*, *even to wash yourself*.

Meanwhile, this fear of stigmatization experienced by family members and this fear of coming out of anonymity also resulted in isolation and withdrawal. Isolation can be chosen and without apparent difficulty; else, a real burden could be felt in their daily lives. In the case of diabetes, as there was no stigma, the barriers to self-management correlated more with the constraints specific to the disease: the dietary barrier associated with dietary restrictions and the guilt of having to impose them, the financial barrier imposed by this diet, the physical barrier associated with the management of hypoglycemia and, finally, the medical barrier imposed by the use of medication, glycemic control, and medical monitoring.

**M2:** *Sometimes I don’t have the money to buy vegetables*, *to eat so I make do with what I can afford so I can eat what I shouldn’t eat*. *When I do it I know I’m not doing it right but how do I do it*?

## Discussion

The three main findings of this study are as follows: (i) the predominance of certain coping strategies over others and that the same strategies were used for HIV and diabetes management, except for associative resources; (ii) medication was still perceived as salvatory, and it was not sufficient or standalone for diabetes management, leading diabetes to be perceived as the severest morbidity and difficult to manage; (iii) the self-management of both HIV and diabetes was not integrated for all participants, as many of them perceived HIV as a separate illness.

### Resources mobilized in the self-management of HIV and diabetes

In this study, emotion-based resources and social support were mobilized more than problem-solving resources, according to Cheng et al.’s classification [38]. Cheng et al looked at coping strategies for chronic conditions and classified them into two types: problem- and emotion-focused strategies.

When a situation is considered “stressful,” individuals make efforts to manage a stressful situation. Emotion-focused coping refers to efforts to manage emotions resulting from polypathology. Problem-focused coping refers to efforts to manage the day-to-day life of the polypatholgy.

Problem-solving resources are primarily represented by medication, as physical activity, diet, or change in lifestyle are not or hardly mentioned; else, they are mentioned negatively in the form of rules that are hard to apply. Conversely, emotion-based resources, such as spirituality, acceptance (fatality and destiny), belief in one’s own strength (positive attitude), and social support, were mentioned very often.

### Problem-solving strategies

The “drug” resource exerted an extremely positive, life-saving image, which is, for many, mobilized from one disease to another. Indeed, compliance with antiretroviral drugs was and is still essential for controlling HIV. There was a time when treatment was simply not available in many countries, especially in the SSA countries from which our participants came from. The only way out was death in the short or long term, which was one of the reasons for the strong stigma attached to HIV infection [39]. The “drug” enabled controlling HIV (the majority of participants had controlled HIV); thus, the same “drug” should also allow the control of diabetes. Nevertheless, the same resource that enabled HIV control was not available for diabetes self-management, as taking oral antidiabetic drugs was only one aspect of the treatment. Diabetes is far more difficult to control than HIV infection, especially as dietary measures are hard to apply.

The more difficult control of diabetes by taking drug alone seems to increase the perception of the severity of diabetes. Yet HIV is, overall, better controlled than diabetes. This result contradicts a 2011 French study on quality of life and perception of severity in HIV/diabetes HIV/diabetes comorbid patients [40]. In this study, 29 patients were asked about the perception of diabetes severity. Among hem, 93% of the patients had well-controlled HIV and only 22% had controlled diabetes. Yet, HIV is perceived as the main and most threatening disease for more threatening disease for more than half of the patients. This study was conducted in 2011 on patients not from SSA, showing that the perception evolves with time and the arrival of ARVs.

### Social support

The social resources mobilized differed from one participant to another, but all mentioned one main resource (family,NGOs, or hospital), which is consistent with a study on the representation of HIV among migrants from SSA [39]. In particular, the relationship of trust in the medical world seems to be acquired with HIV and then reused for diabetes. By following the doctor’s rules and medicines, the disease can be fought. Moreover, we found that the relationship with the infectious disease specialist was very close for many, sometimes almost to the point of deification of their referent.

Whether the doctor–patient relationship mentioned above have a specificity among patients from SSA remains unclear. The idea of the physician as a savior and holder of knowledge is, perhaps, more deeply rooted in some cultures; however, the therapeutic physician–patient relationship in the management of HIV is especially strong and paramount in HIV-infected patients from SSA, as also shown in many studies [41, 42]. However, this result goes against the grain of the empowerment advocated by the scientific community and citizens, which leads patients to autonomy in health and better management of their illness [43].

In this specific population of participants from SSA treated for HIV, we assumed that this relationship of trust between the cared-for and the caregiver could either be necessary and sufficient in the management of their pathology or be a prerequisite to enable empowerment and arrive at a shared medical decision. Moreover, the general practitioner (GP) was not mentioned as a participant or coordinator of care, undermining these two essential roles of the GP in polypathology.

The importance of NGOs resources in our sample corroborates the HIV literature. A retrospective study in Mozambique examining 1413 patients demonstrated a correlation between the absence of NGOs resources and nonadherence to ARV [44]. While this resource is essential in HIV self-management, it is not used in diabetes self-management. Although we did not find any literature on this subject, we formulated a few hypotheses: (i) patients miss this resource because they are not aware of it; (ii) the history of previous chronic disease, such as HIV, renders this resource cumbersome and redundant, preventing new recourse; (iii) the NGOs resource is often present to compensate for the family resource in the context of secrecy, but this same resource is no longer mobilized in the context of diabetes, an easily “dictated” disease. Although not widely used in our study, the value of NGOs for diabetes self-management has been well demonstrated in the literature. They have a major impact on improving clinical, lifestyle and psychosocial outcomes in people with Type 2 diabetes [45, 46].

This type of support is used for HIV and not for diabetes which could be an argument for incorporation of comorbidity care into community-based approaches to support people living with HIV.

### Emotion-based strategies

Spirituality plays a key role, and it was cited as a common resource in HIV and diabetes in this study. Regardless of the religion (Muslim or Catholic), “God” gives meaning to the “disease” in terms of its cause, its evolution, and the means of accepting and fighting it, corroborating a Belgian study on a migrant population from SSA, where 46 patients (2 Muslims and 44 Christians) were asked about the role of religion in their HIV management; as in our study, a majority described God as a major factor impacting on their treatment and on the evolution of their pathology [47].

This resource, integrated with “emotion-based strategy” according to Cheng et al. [38], seemed to have a marked cultural dimension. Indeed, Leach and Schoenberg [48] suggested a clear difference in the expression and use of this resource between the two populations (Black Americans and White Americans). In addition, support from “God” and the church was central to the self-management of polypathology in the Black American population; one of the explanations for this was Taylor et al.’s model of the historical intertwining of religion and civil society in this population in many areas, including health [49].

In parallel with the stigma present within their religious community, this resource might raise questions about the need to intensify stigma prevention in this area. To date, numerous HIV prevention activities in different religious communities (mosques or churches) have been executed in France [50, 51] with a beneficial effect in terms of therapeutic education and control [52]. Concerning diabete, some church-based diabetes self-management education program have been described, especially for African-Americans with type 2 diabetes, which contribute to improve diabetes care in this population [52]. Indeed, few links exist between health professionals and these religious communities, which could play a major role in the quality of life related to HIV in patients from SSA.

Thus, our results suggested that, despite the medical progress and the progression of HIV toward a chronic pathology, the individual representation is progressing less quickly and remains closely related to the community representation because of the emotional dimension, which would lead an individual to mobilize additional “emotion-based” resources.

### Social legitimacy as a patient with a diagnosis of diabetes and severity that changes sides

For most participants, HIV remained a separate pathology, even within polypathology, primarily because of the stigma that HIV generates and the representation dominated by a certain link with death. In addition, our study aligns with a study conducted in Burkina Faso on 219 PLWHA on the different forms of stigmatization related to HIV [53]. Accordingly, several forms of stigmatization were highlighted: self-stigmatization (46%), stigmatization in interpersonal relationships (40%), and stigmatization in health services (11%) [53]. In our sample, self-stigma and interpersonal stigma were the main issues, and none of the participant mentioned stigma in the medical environment.

The diagnosis of diabetes is, for many, an entry into a “dictable” disease, which we can talk to others about, and participants acquire the social status of “being sick.” Once HIV is accepted and controlled (easily by medication), the severity shifts to diabetes. In our study, this perception of diabetes severity was objectified by less controlled diabetes than HIV. Previously, an American study on polypathology and HIV [28] in a predominantly Black American population reported that HIV was also perceived as less severe than diabetes, and the perception of diabetes as the most serious condition seemingly correlated with the integration of their medical discourse and the representation of diabetes through amputation. As with HIV, numerous studies demonstrated a close relationship between the representation of diabetes and involvement in this disease [54].

We found no study specifically on the representation of type 2 diabetes in migrants from SSA living in France. A qualitative English study examined the correlation between disease representation and diabetes management in patients from England and SSA and showed that diabetes self-management was hampered if diabetes was perceived as a disease with severe consequences in both populations [55]; our study confirms this result. As mentioned earlier, diabetes seems to be perceived as a hereditary disease with severe consequences that patients cannot control by themselves; thus, it is essential to change the representations of diabetes and provide access to more problem-based resources for the management of diabetes in particular.

### Implications for the care pathway in polypathology when one of the pathologies is HIV

Numerous studies in several countries mentioned the barrier to the organization of the healthcare system for patients with several diseases. An American qualitative study on polypathology and HIV reported difficulty in communication among practitioners as one of the barriers to managing polypathology [37]. Another study on barriers and resources to manage HIV and diabetes in Cape Town highlighted the need for integrated services that included management of all their conditions, therapeutic education materials, and improved information from practitioners about their condition [56]; our results contradict these findings. When a diabetologist or a GP was present, communication among doctors was not mentioned as a barrier, and no participant was bothered by certain contradictions among the different practitioners, which probably also related to a low level of literacy in its critical dimension. Indeed, other studies reported a significant discomfort related to contradictory medical discourses in populations with high literacy levels [57, 58].

This quasi-blind trust would be a specificity of our sample related to the onset of disease by HIV, which could create a particular doctor–patient relationship. Indeed, our study involved participants who already had a referral infectious diseases specialist with whom a bond of trust had often been established. Had our study included only participants who were initially diabetic and then diagnosed with HIV, the results could have been different. Furthermore, the issue of participants’ confidence in the French healthcare system and in their health professionals is a key resilience factor. A resilience factor can be described as the ability to achieve a goal despite a given ordeal [59].

A study in Ghana investigated the resilience factors HIV-infected patients that enabled them to engage and remain engaged in their care [57], reporting three factors, one of which was the appropriate level of trust in their health professionals. This doctor–patient relationship seems to be transposable from one pathology to another, which has implications in terms of the care pathway. The GP, who is supposed to play the main role in coordinating the care pathway, could play a much more central role than at present. If the diagnosis of these pathologies was done by a GP with more episodic follow-up by specialists, the trust could be built between the GP and these patients. Moreover, the perception of HIV could also be maintained by a predominantly hospital-based approach. Furthermore, the involvement of the GP from the moment of the diagnosis would, perhaps, make it possible to fight against stigmatization by demystifying the monitoring of PLWHA, followed by real integrated care.

### Strengths and limitation

First, some fatigue with the redundancy of the questions might have arisen because of the chronology of the questions asked about diabetes following those about HIV. In fact, the guide mainly used the same open-ended questions for HIV and then for diabetes This may have masked some resources or difficulties specific to diabetes. Second, this study is a single-center study, which is not representative of all models of doctor–patient relationships. Third, our study included HIV-infected participants with diabetes; only one participant had uncontrolled HIV and 5 had uncontrolled diabetes. As markers or not of involvement in both conditions, the results could be influenced by this recruitment bias and could be different if the study included participants in whom both conditions were controlled. Finally, only one participant was treated with insulin, which mainly limited the results of the study to non-insulin-dependent diabetes. Although necessary to analyze the self-management of both diseases, the age of the two diseases could constitute a memory bias. Hence, further studies are warranted to overcome these limitations and validate our findings.

We however believe that our study, using a qualitative method adapted to an in-depth exploration of participants’ disease experience, brings an important insight to a topic insufficiently studied in France, which also may be transposable in other countries.

Furthermore, the fact that our samples included participants whose settlement in France dated back at least 12 years ago allowed to remove some traditional barriers to self-management such as financial barriers and reveal other barriers and facilitators.

## Conclusions

The self-management of PLWHA with diabetes will be all the more successful if patients and carers move in the same direction, having identified the individual levers on which to rely to attain their objectives. Caregivers must understand the complexity of polypathology related to the socioeconomic and cultural specificities of this population and identify for each patient the main resource on which both doctor and patient can rely. Patients must be able to be actors in their care if spaces are create to allow them to be empowered. The split organization of care for both diseases seems satisfactory for patients because of the persistent secrecy surrounding HIV. The trust established with the doctor who informs the diagnosis and then “saves” the patient could be a major resilience factor for this population. However, integrated multidisciplinary care could benefit patients if the current pathways are transformed, especially by consolidating the role of the care coordinator by the GP.

## Supporting information

S1 Checklist COREQ checklist. (PDF)

S1 Annex Interview guide. (DOCX)
