# Supplementary material for: Perceptions of multiple chronic conditions and coping strategies among migrants from Sub-Saharan Africa living in France with diabetes mellitus and HIV: An interview-based qualitative study
Source: PLoS One. 2023 Jun 2;18(6):e0284688. doi: 10.1371/journal.pone.0284688 (PMC10237665; doi:10.1371/journal.pone.0284688)
Supplement: S1 Annex — (DOCX) [file pone.0284688.s002.docx]

**Annex 1. Interview guide.**

1. HIV self-management

Can you tell us about your experience of being told you are HIV-positive?

What does living with HIV mean to you?

Can you tell us what HIV means to you?

Can you tell us what has changed in your life?

Have you encountered any difficulties since you were diagnosed as HIV-positive, and if so, which ones?

What resources did you use to deal with it?

Can you tell us about your experience with ARV treatment?

Can you give us an example of a noncompliance circumstance and its context?

1. Diabetes self-management

Can you tell us about your experience when you were diagnosed with diabetes?

What did you think about immediately after the diagnosis?

What were the differences with HIV?

What does diabetes mean to you?

Can you tell us what has changed in your life?

What resources do you have for good diabetes control?

Can you tell us about your experience with diabetes treatment?

Can you give us an example of a noncompliance circumstance and its context?

1. Self-management of polypathology

Can you tell us about your experience of living with two chronic diseases?

Which disease do you know best?

Which disease do you consider the most serious?

Which disease is the most difficult to live with?

Of the resources you have mobilized for HIV, have you used any for diabetes?

On the contrary, is there anything that is not applicable for diabetes?

Do you feel that diabetes has helped you with HIV adherence?

Can you give us an example of a common difficulty?

Can you tell us about your family’s experience with polypathology?

1. Care in the healthcare system

How are you treated for HIV? Consultations? Day hospital? FCT? What out-of-hospital resources do you use?

What about diabetes?

Are you satisfied with the organization of medical care for these two diseases?

Is there any information that is repeated from one consultation to another?

When you go for a diabetes consultation, are you told about HIV? And when you go to see a doctor about HIV, do they tell you about diabetes?

Do you have any suggestions for improving the current organization of your care?

1. Sociodemographic sheet

Age, ethnicity, marital status, number of children? Country of birth? Date of arrival in France?

Date, reason, context, and course of the migration?

Lifestyle on arrival in France, community or family or NGOs.

Work (permanent, fixed-term, temporary, looking for work, homemaker, student), financial resources (range of monthly income)

Literacy level: reads French fluently, needs help to read, does not read at all

Type of housing: tenant/owner/landlord
